# Mutations in the cytoplasmic iron-sulfur assembly protein CIAO1 cause neuromuscular deficits

**DOI:** 10.1172/JCI182474

**Published:** 2024-06-17

**Authors:** Puneet Opal

Iron-sulfur clusters emerged over three billion years ago in microbes, allowing the eventual evolution of biological processes such as respiration, photosynthesis, and nitrogen fixation (1). In eukaryotes, iron-sulfur proteins contribute to cellular activities from DNA replication and repair to heme synthesis and electron transfer in mitochondria. Despite these diverse functions, the biochemical pathways that synthesize and assemble iron-sulfur clusters onto client proteins are highly conserved.

Studies in yeast and mammalian cells have determined that mitochondrial iron-sulfur proteins are assembled from a core iron-sulfur complex (ISC) involving multiple components. The cysteine desulfurase NFS1 (stabilized by the accessory proteins ISD11/LYRM4 and acyl carrier protein ACP) converts cysteine to alanine to generate a sulfur group, which is then transferred to a cysteine of a scaffold protein, ISCU, with iron provided by the iron donor frataxin (1). Mutations in most of these proteins, along with accessory cluster carriers and chaperones, have been associated with human diseases that manifest as mitochondrial dysfunction (2). Some of the diseases feature anemia as a major component, reflecting the role of these clusters in erythrocytes.

Iron-sulfur enzymes localized outside mitochondria require an additional cytosolic iron-sulfur assembly (CIA) complex (3, 4). This complex comprises CIAO1, MMS19, and FAM96B, which add iron-sulfur clusters to client proteins in the nucleus or cytosol. Their contribution to human health is far less studied, in part because no genetic diseases have been described — until now.

In this issue of the *JCI*, Maio et al. describe four unrelated individuals with biallelic mutations in *CIAO1* (5). These patients experienced childhood or juvenile onset of limb, facial, and bulbar weakness, accompanied by respiratory weakness and elevated muscle enzymes. The authors describe variable learning and behavioral problems, along with anemia and constipation. Three patients were compound heterozygous for missense variants in *CIAO1*, and one patient has a missense variant on one allele and a deletion on the other. Computational analyses predicted the missense variants to be pathogenic, while the deletion was expected to generate an out-of-frame transcript undergoing nonsense-mediated decay.

The researchers investigated the biogenesis and activity of client enzymes dependent on Fe-S clusters from the CIA complex. They demonstrated a reduction in the levels of several iron-sulfur proteins and in the biosynthesis of two paradigmatic enzymes, POLD1 and DPYD, which are involved in DNA replication and the degradation of pyrimidine bases, respectively. Importantly, lentivirus-mediated transduction with wild-type *CIAO1* rescued the phenotypic abnormalities in patient-derived cells (5).

One notable aspect of CIAO1 deficiency is its overlap with mitochondrial myopathies, including mitochondrial ultrastructural and functional deficits. This finding suggests that extramitochondrial iron-sulfur proteins influence mitochondrial function (5). Further investigation into additional mutations in CIAO1 and other CIA complex members will likely shed light on this puzzling issue and the role of extramitochondrial iron-sulfur proteins more generally in health and disease.

## Version 1 06/17/2024

Electronic publication
